# The conserved threonine-rich region of the HCF-1_PRO_ repeat activates promiscuous OGT:UDP-GlcNAc glycosylation and proteolysis activities

**DOI:** 10.1074/jbc.RA118.004185

**Published:** 2018-09-17

**Authors:** Vaibhav Kapuria, Ute F. Röhrig, Patrice Waridel, Fabienne Lammers, Vladimir S. Borodkin, Daan M. F. van Aalten, Vincent Zoete, Winship Herr

**Affiliations:** From the ‡ Center for Integrative Genomics, University of Lausanne, 1015 Lausanne, Switzerland,; the § Molecular Modelling Group, SIB Swiss Institute of Bioinformatics, Lausanne 1015, Switzerland,; the ¶ Protein Analysis Facility, Center for Integrative Genomics, Faculty of Biology and Medicine, University of Lausanne, 1015 Lausanne, Switzerland,; the ‖ Centre for Gene Regulation and Expression, School of Life Sciences, University of Dundee, Dundee DD1 5EH, Scotland, United Kingdom, and; the ** Department of Fundamental Oncology, Ludwig Lausanne Branch, Faculty of Biology and Medicine, University of Lausanne, 1066 Epalinges, Switzerland

**Keywords:** O-GlcNAcylation, O-linked N-acetylglucosamine (O-GlcNAc) transferase (OGT), post-translational modification (PTM), enzyme mechanism, glycobiology, host-cell factor-1

## Abstract

*O*-Linked GlcNAc transferase (OGT) possesses dual glycosyltransferase–protease activities. OGT thereby stably glycosylates serines and threonines of numerous proteins and, via a transient glutamate glycosylation, cleaves a single known substrate—the so-called HCF-1_PRO_ repeat of the transcriptional co-regulator host-cell factor 1 (HCF-1). Here, we probed the relationship between these distinct glycosylation and proteolytic activities. For proteolysis, the HCF-1_PRO_ repeat possesses an important extended threonine-rich region that is tightly bound by the OGT tetratricopeptide-repeat (TPR) region. We report that linkage of this HCF-1_PRO_-repeat, threonine-rich region to heterologous substrate sequences also potentiates robust serine glycosylation with the otherwise poor *R*_p_-αS-UDP-GlcNAc diastereomer phosphorothioate and UDP-5S-GlcNAc OGT co-substrates. Furthermore, it potentiated proteolysis of a non-HCF-1_PRO_-repeat cleavage sequence, provided it contained an appropriately positioned glutamate residue. Using serine- or glutamate-containing HCF-1_PRO_-repeat sequences, we show that proposed OGT-based or UDP-GlcNAc–based serine-acceptor residue activation mechanisms can be circumvented independently, but not when disrupted together. In contrast, disruption of both proposed activation mechanisms even in combination did not inhibit OGT-mediated proteolysis. These results reveal a multiplicity of OGT glycosylation strategies, some leading to proteolysis, which could be targets of alternative molecular regulatory strategies.

## Introduction

GlcNAc transferase (OGT) ^4^ has an unusual dual glycosyltransferase–protease activity (1). It is implicated in numerous intracellular signaling pathways through *O*-linked addition of a single GlcNAc moiety to acceptor serines or threonines in target proteins; these modifications are reversible by the enzymatic activity of *O*-GlcNAcase. In addition to this “canonical” serine-threonine glycosyltransferase activity, OGT has a special protease activity in which it cleaves a very specific substrate—the 26-amino acid HCF-1_PRO_ repeat of the cell proliferation host-cell factor HCF-1—at a key glutamate residue at position 10 (Glu-10) of the HCF-1_PRO_ repeat (1, 2).

The proteolytic activity results from a second “noncanonical” glycosyltransferase activity—to date specific to the HCF-1_PRO_ repeat—by which OGT *O*-GlcNAcylates the Glu-10 residue, leading to autoproteolysis (3). Curiously, glycosylation of a glutamate residue in a non-OGT–related context is sufficient for spontaneous backbone cleavage (4). Thus, the HCF-1_PRO_ repeat appears to be a very specific OGT cleavage substrate because it is able to induce OGT to *O*-GlcNAcylate the Glu-10 residue. Indeed, the HCF-1_PRO_ repeat interacts intimately with OGT across much of its length, favorably positioning the Glu-10 residue adjacent to the UDP-GlcNAc for glycosylation (2). The close relationship of glutamate and serine/threonine glycosylation is substantiated by the finding that a Glu-to-Ser substitution (E10S) changes the HCF-1_PRO_ repeat from a glutamate-directed cleavage substrate into a serine-directed stable glycosylation substrate (2).

Here, we examine the glycosylation and cleavage activities of OGT on HCF-1_PRO_ repeat–related and –nonrelated substrates and find that position 10 of the HCF-1_PRO_ repeat confers special enzymatic OGT glycosylation properties on substrates leading to different outcomes: canonical serine glycosylation or noncanonical glutamate-induced proteolysis.

## Results

To study the canonical serine and threonine glycosylation and noncanonical glycosyl glutamate–induced cleavage activities of OGT, we used human casein kinase 2 (CK2) and HCF-1_PRO_-repeat substrates, respectively, using both native and recombinant proteins as well as synthetic peptides. Protein substrates were used for immunoblot analysis with both GlcNAc and protein substrate–specific antibodies, whereas peptides were used for MS analysis. Additionally, to assess UDP-GlcNAc co-substrate requirements, we also used three different oxygen-to-sulfur–substituted UDP-GlcNAc co-substrates—the two *R*_p_-αS-UDP-GlcNAc and *S*_p_-αS-UDP-GlcNAc diastereomer phosphorothioate analogs (5) and the pyranose ring–modified UDP-5S-GlcNAc (6).

### The HCF-1_PRO_-repeat E10S substrate displays relaxed UDP-GlcNAc co-substrate requirements

**Figure 1.**
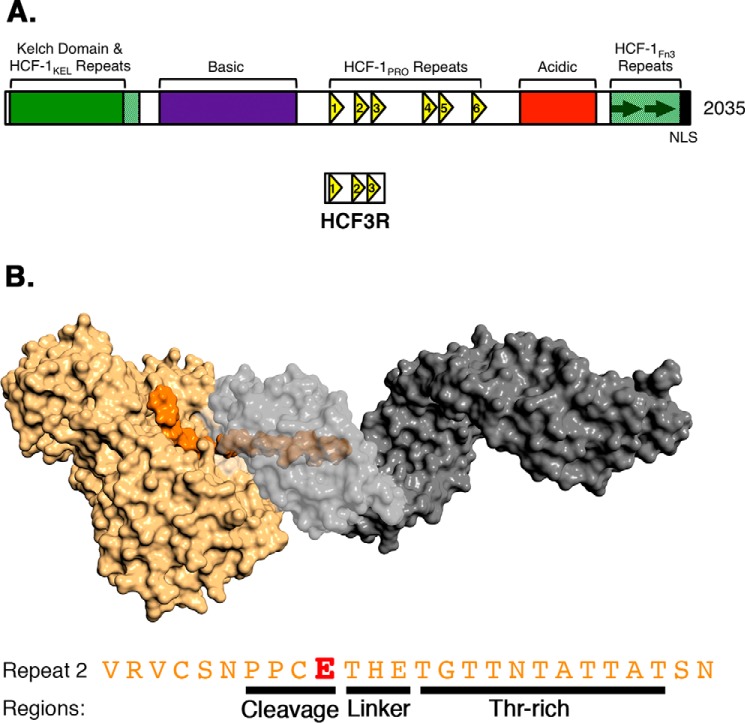
**Human HCF-1, OGT, and HCF-1_PRO_ repeat.** *A*, domain representation of human HCF-1. The centrally located HCF-1_PRO_ repeats are shown as *yellow triangles*. The HCF3R substrate contains HCF-1_PRO_ repeats 1–3. *B*, depiction of HCF-1_PRO_ repeat 2 and OGT based on crystal structures 4N3B and 1W3B. The HCF-1_PRO_ repeat (*orange*) interacts with the OGT catalytic domain (*tan*) as well as the OGT TPR domain (*gray*). TPR residues 313–463 are *transparent* to show the internal occupancy of the HCF-1_PRO_-repeat 2 Thr-rich region. The amino acid sequence of HCF-1_PRO_ repeat 2, further classified into cleavage (residues 7–10), linker (residues 11–13), and Thr-rich (residues 14–24) regions, is shown *below. E* (*red*), site of proteolytic cleavage.

**Figure 2.**
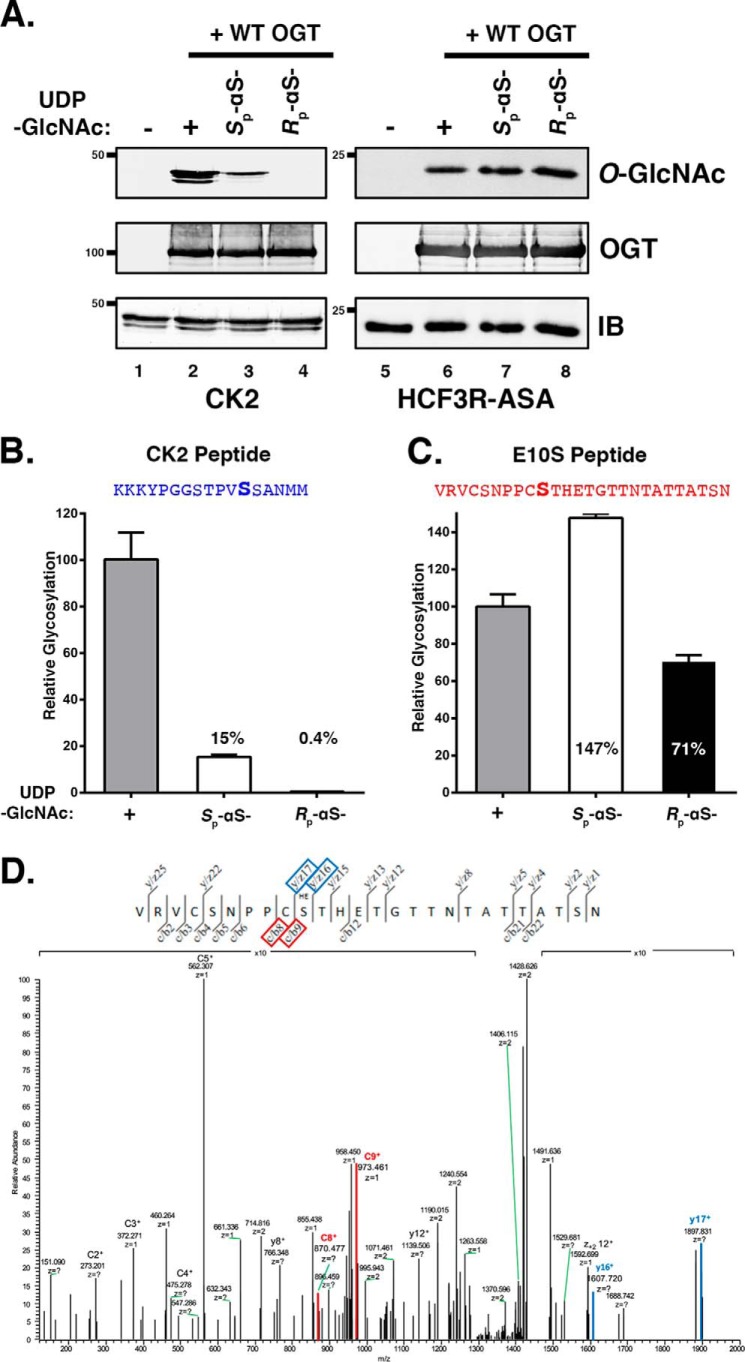
**The HCF-1_PRO_-repeat E10S substrate displays relaxed UDP-GlcNAc co-substrate requirements.** *A*, *in vitro* substrate glycosylation assays were performed using CK2 (*lanes 1–4*) and HCF3R-ASA proteins (*lanes 5–8*). Substrates were incubated without OGT (*lanes 1* and *5*) or with OGT and either UDP-GlcNAc (*lanes 2* and *6*), *S*_p_-αS-UDP-GlcNAc (*lanes 3* and *7*), or *R*_p_-αS-UDP-GlcNAc (*lanes 4* and *8*). Substrate glycosylation was detected using RL2 anti-*O*-GlcNAc antibody. Anti-OGT, -CK2, and -His antibodies were used to detect OGT, CK2, and HCF3R-ASA proteins, respectively. *B* and *C*, *in vitro* peptide glycosylation assays were performed using CK2 (*B*) and HCF-1_PRO_-repeat 2 E10S (*C*) peptides. Peptides were incubated with WT OGT and either UDP-GlcNAc, *S*_p_-αS-UDP-GlcNAc, or *R*_p_-αS-UDP-GlcNAc, as indicated. Peptide glycosylation was detected using LC-MS. *O*-GlcNAcylation of the CK2 and E10S peptides in different reactions was normalized to the *O*-GlcNAcylation activity with UDP-GlcNAc: 6.3 × 10^7^ and 1.3 × 10^9^, respectively. *D*, EThcD analysis of the *R*_p_-αS-UDP-GlcNAc E10S glycopeptide product. *Red* and *blue boxed* products indicate the N-terminal c/b8 and c/b9, and C-terminal y/z17 and y/z16 ions, respectively, that flank the glycosylated serine residue. *Error bars*, S.D.

Fig. 1 shows (i) the general structure of HCF-1 (*A*) and (ii) a surface representation of the OGT-HCF-1_PRO_-repeat complex with the OGT catalytic domain in *tan* and the 13.5 tetratricopeptide-repeat (TPR) domain in *gray*, together with the HCF-1_PRO_-repeat 2 sequence (*orange*) with regions identified (*B*). Human HCF-1 contains six cleavable HCF-1_PRO_ repeats (HCF-1_PRO_ repeats 1–6) (7, 8). The HCF-1_PRO_ repeat–based protein substrates used here were variants of the previously described HCF3R bacterial expression construct (2), which contains HCF-1_PRO_ repeats 1–3 (Fig. 1*A*). To retain only one active HCF-1_PRO_ repeat, HCF-1_PRO_ repeats 1 and 3 were inactivated by an E10A glutamate-to-alanine substitution, creating the so-called HCF3R-AEA substrate. For HCF-1_PRO_-repeat E10S *O*-GlcNAcylation, we prepared the related noncleavable HCF3R-ASA construct (see Table S2 for sequences). As shown in Fig. 2*A*, like the canonical CK2 OGT substrate, HCF3R-ASA is also an effective OGT *O*-GlcNAcylation substrate (compare *lanes 1* and *2* with *lanes 5* and *6*).

Consistent with previous results using other substrates (5, 9), CK2 substrate *O*-GlcNAcylation by OGT was maintained, albeit with reduced efficiency, when *S*_p_-αS-UDP-GlcNAc was utilized, whereas no *O*-GlcNAcylated CK2 protein was observed in the presence of *R*_p_-αS-UDP-GlcNAc (Fig. 2*A*, compare *lanes 3* and *4*). In strong contrast, reactions using the HCF3R-ASA substrate and either of the two UDP-GlcNAc diastereomers as co-substrate yielded similar robust *O*-GlcNAcylation activities (Fig. 2*A*, *lanes 7* and *8*). The strong HCF3R-ASA glycosylation with *R*_p_-αS-UDP-GlcNAc was unexpected.

To study this unexpected activity further, we adopted a peptide substrate coupled with an HPLC-tandem MS (LC-MS/MS) approach. By using peptide substrates and MS, we could easily manipulate well-defined peptide sequences as well as compare the number and site(s) of glycosylation in the resulting reaction products. As CK2 and HCF-1_PRO_-repeat representatives, we used the previously described CK2-derived peptide NH_2_-KKKYPGGSTPVSSANMM-COOH (10) (serine acceptor underlined) and an HCF-1_PRO_ repeat 2–derived E10S peptide NH_2_-VRVCSNPPCSTHETGTTNTATTATSN-COOH (E10S position underlined) as OGT substrates. Consistent with the immunoblotting results in Fig. 2*A*, we observed *O*-GlcNAcylation of the CK2 peptide by OGT with UDP-GlcNAc and with *S*_p_-αS-UDP-GlcNAc, albeit at reduced levels, whereas *R*_p_-αS-UDP-GlcNAc was essentially inactive (Fig. 2*B*). Also consistent with the immunoblotting results, we observed *O*-GlcNAcylation of the E10S peptide by OGT with any of the three UDP-GlcNAc and *S*_p_- and *R*_p_-αS-UDP-GlcNAc co-substrates (Fig. 2*C*). A time course showed that the two diastereomer UDP-GlcNAc substrates had parallel rates of glycosylation (Fig. S1).

To verify the precise status of E10S peptide glycosylation, we performed electron transfer dissociation analysis of the *R*_p_-αS-UDP-GlcNAc E10S peptide product. As shown in Fig. 2*D*, the product is uniquely glycosylated at the E10S position. Thus, the E10S peptide relaxes the molecular constraints on UDP-GlcNAc–related co-substrates for OGT glycosyltransferase activity.

### The HCF-1_PRO_-repeat threonine-rich region is responsible for the relaxed E10S peptide UDP-GlcNAc co-substrate requirements

To identify the properties of the E10S peptide responsible for its unusual relaxed UDP-GlcNAc co-substrate requirements, we compared the co-substrate requirements of the CK2 and E10S peptides by preparing two chimeric (Ch) peptides. As shown in Fig. 1*B*, the HCF-1_PRO_ repeat has been divided into three regions: cleavage (PPCE) and threonine-rich (TGTTNTATTAT) regions held together by a flexible three amino acid (THE) linker segment (9). In the OGT-bound WT HCF-1_PRO_-repeat substrate, the Glu-10–containing cleavage region lies beside the UDP-GlcNAc co-substrate in the OGT catalytic domain, and the threonine-rich region binds the TPR domain anchoring the HCF-1_PRO_ repeat in OGT (2). In the E10S HCF-1_PRO_-repeat substrate, the WT HCF-1_PRO_-repeat cleavage region is converted into a “glycosylation region.”

**Figure 3.**
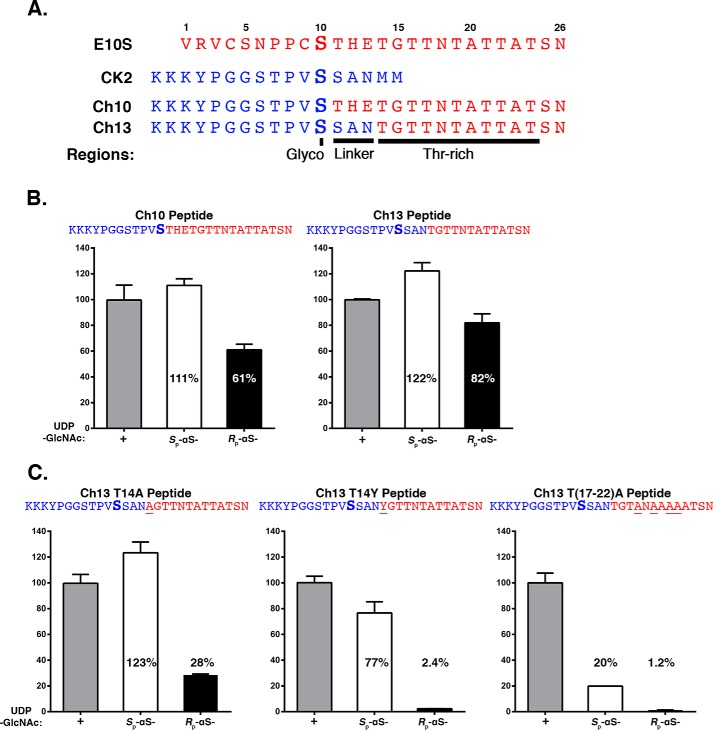
**The HCF-1_PRO_-repeat threonine-rich region is responsible for the relaxed E10S peptide UDP-GlcNAc co-substrate requirements.** *A*, amino acid sequences of native E10S and CK2 peptides as well as two chimeric peptides derived from CK2-E10S fusion (Ch10 and Ch13) are shown. The serine for glycosylation (*Glyco*) is depicted in *boldface type*. The linker and Thr-rich region are indicated. *B* and *C*, *in vitro* peptide glycosylation assays were performed using Ch10 and Ch13 chimeric peptides (*B*) and Ch13 T14A, T14Y, and T(17–22)A mutant peptides (*C*). Peptides were incubated with WT OGT and either UDP-GlcNAc, *S*_p_-αS-UDP-GlcNAc, or *R*_p_-αS-UDP-GlcNAc, as indicated. Peptide glycosylation was detected using LC-MS. *O*-GlcNAcylation of the different peptides was in each case normalized to *O*-GlcNAcylation activity with UDP-GlcNAc as follows: Ch10, 3.2 × 10^8^; Ch13 WT, 1.6 × 10^8^; Ch13 T14A, 3.6 × 10^8^; Ch13 T14Y, 2.0 × 10^8^; and Ch13 T(17–22)A, 1.5 × 10^7^. *Error bars*, S.D.

As shown in Fig. 3*A*, the first chimeric peptide, called Ch10, replaced the N-terminal E10S HCF-1_PRO_-repeat glycosylation region (residues 1–10) with the corresponding CK2 peptide sequence (residues 1–12). The second, called Ch13, replaced both the glycosylation region and the THE linker (residues 1–13) with the corresponding CK2 peptide sequence (residues 1–15). These two chimeric peptides displayed co-substrate glycosylation selectivity similar to the HCF-1_PRO_-repeat E10S peptide, being also readily active with the *R*_p_-αS-UDP-GlcNAc co-substrate. Thus, neither the E10S HCF-1_PRO_-repeat glycosylation region nor the THE linker is essential for its relaxed co-substrate requirements.

These results led us to focus on the importance of the threonine-rich region, for which we created three Ch13 peptide–based mutants, called Ch13-T14A, Ch13-T14Y and Ch13-T(17–22)A. In Ch13-T14A and Ch13-T14Y, we replaced position Thr-14, the first residue of the threonine-rich region (see Fig. 3*A*) and a residue critical for HCF-1_PRO_-repeat cleavage (11), either conservatively with alanine (Ch13-T14A) or radically with tyrosine (Ch13-T14Y). In Ch13-T(17–22)A, we replaced four conserved threonine residues with alanine, a mutation that severely disrupts OGT binding to the HCF-1_PRO_ repeat (1).

Fig. 3*C* shows the results of peptide glycosylation assays with UDP-GlcNAc and the *S*_p_- and *R*_p_-αS-UDP-GlcNAc diastereomers and these three threonine-rich region mutants. For each peptide, the results are normalized to the activity of UDP-GlcNAc for that peptide (see the legend to Fig. 3). The Ch13 parental and Ch13-T14A and Ch13-T14Y mutant peptides displayed similar activities with UDP-GlcNAc, whereas the radical threonine-region Ch13-T(17–22)A mutant was more than 10-fold less active. These results suggest that for normal UDP-GlcNAc–induced glycosylation, the threonine region is important because the Ch13-T(17–22)A mutant displays little activity and yet is not easily inactivated, as the Ch13-T14A and Ch13-T14Y mutants remain active in these assays. Indeed, the Ch13-T(17–22)A mutant showed activities similar to the CK2 peptide: low overall UDP-GlcNAc utilization and strong selectivity against the *R*_p_-αS-UDP-GlcNAc diastereomer (compare Fig. 2*B* with Fig. 3*C*). In contrast, the Ch13-T14A and Ch13-T14Y mutants have high UDP-GlcNAc utilization and yet curiously display increasing discrimination between the two *S*_p_- and *R*_p_-αS-UDP-GlcNAc diastereomers, whereas the *R*_p_-αS-UDP-GlcNAc diastereomer displays little if any activity with the Ch13-T14Y mutant chimeric peptide. Thus, the juxtaposition of the HCF-1_PRO_-repeat threonine-rich region to the CK2 glycosylation site in the Ch13 chimeric peptide has two distinct and separable effects on CK2 sequence glycosylation: (i) it stimulates glycosylation and (ii) relaxes the constraints on the use of the *R*_p_-αS-UDP-GlcNAc co-substrate for OGT serine glycosylation.

### Molecular dynamic simulations of OGT/UDP-GlcNAc–peptide substrate interactions

**Figure 4.**
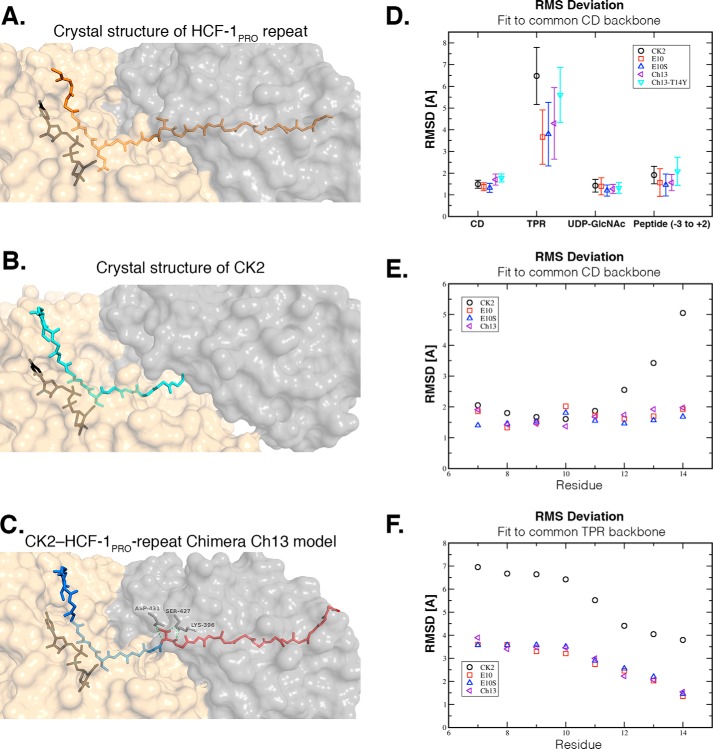
**Structural representations and MD simulations of OGT-UDP-GlcNAc–peptide complexes.** *A–C*, representations of the structures of HCF-1_PRO_ repeat 2 (PDB code 4N3B) (*A*), CK2 (PDB code 4GYY) (*B*), and chimeric Ch13 peptide (homology model) (*C*) to the OGT (TPR domain (*gray*) and catalytic domain (*beige*)). TPR domain residues that make critical backbone and side-chain interactions with the Thr-14 residue of Ch13 are shown in *C. D*, RMSDs of different parts of the complexes from their initial structures observed during MD simulations. Based on structural superimposition of the backbone of the catalytic domain of OGT (*CD*), the RMSD of the OGT catalytic domain, the OGT TPR domain, the UDP-GlcNAc sugar donor, and the central part of the bound peptide are shown from *left* to *right*. The central part of the peptide includes three amino acids downstream and two upstream of the modified residue. Simulated systems are OGT bound to CK2 (*black circle*), HCF-1_PRO_-repeat 2 Glu-10 (*red square*), HCF-1_PRO_-repeat 2 E10S (*blue triangle*), and the chimeric Ch13 peptide (WT (*purple triangle*) and T14Y mutant (*turquoise triangle*)). *E*, RMSD of the central six amino acids of each peptide from their initial structure after structural superimposition of the backbone of the TPR domain of OGT. Peptides that lack the HCF-1_PRO_-repeat Thr-rich region (CK2, *black circle*) show larger deviation movements than those that possess the HCF-1_PRO_-repeat Thr-rich region (Glu-10, E10S, and Ch13). *F*, RMSD of the central six amino acids of each peptide from their initial structure after structural superimposition of the backbone of the TPR domain of OGT. Peptides that lack the HCF-1_PRO_-repeat Thr-rich region (CK2, *black circle*) show larger deviations than those that possess the HCF-1_PRO_-repeat Thr-rich region (Glu-10, E10S, and Ch13). *Error bars*, S.D.

The aforementioned studies reveal a striking difference in activity of the CK2 and HCF-1_PRO_-repeat E10S substrates and that the CK2 substrate can be converted to an HCF-1_PRO_-repeat E10S-like substrate by fusion to the HCF-1_PRO_-repeat threonine-rich region. Given this unusual activity spectrum, we turned to computational methods to investigate how these different substrates might interact with OGT and UDP-GlcNAc. As shown in Fig. 4*C*, we generated an initial structure of the chimeric Ch13 peptide sequence by homology modeling, using the X-ray diffraction–determined structures of the parental HCF-1_PRO_ repeat and CK2 (Fig. 4, *A* and *B*) (2) as templates. This approach yielded an OGT-bound Ch13 peptide structure very similar to the CK2 crystal structure of Lazarus *et al.* (12) (Fig. 4*B*) in the N terminus up to the serine-acceptor residue (root mean square deviation (RMSD) = 1.25 Å) and a structure similar to the WT HCF-1_PRO_-repeat peptide in the threonine-rich region (RMSD = 0.90 Å). The SAN linker region of the CK2 sequence adopts a conformation close to the linker region of the HCF-1_PRO_ repeat.

To test the relevance of the modeled OGT-bound chimeric Ch13 peptide structure and to compare the molecular properties of the three OGT-bound peptides, we turned to molecular dynamic simulations as shown in Fig. 4 (*D–F*). Fig. 4*D* shows the relative movements of the TPR and catalytic domains as well as of UDP-GlcNAc and of the peptide glycosylation region (−3 to +2 of the serine acceptor at position 0) with respect to the OGT catalytic domain X-ray structure. As expected, we observed little relative movement of the catalytic domain and UDP-GlcNAc moieties when bound to five different peptide substrates: CK2, HCF-1_PRO_-rep 2 WT (Glu-10) and E10S mutant, and chimeric Ch13 and Ch13-T14Y peptides.

As described previously (10), we observed significant TPR-domain movements relative to the catalytic domain (Fig. 4*D*). However, relative TPR-domain movements of OGT bound to the two HCF-1 and chimeric Ch13 peptides were more constrained than those of the CK2 peptide. These results suggest that peptides binding to both the catalytic domain and the TPR domain restrict inter-TPR and catalytic domain movements. The mutant Ch13-T14Y peptide-bound OGT displayed less constrained inter-TPR and catalytic domain movements, similar to the CK2-bound OGT. These latter results are consistent with an unstable TPR-Ch13-T14Y interaction observed in the molecular dynamic simulations (data not shown) and the aforementioned similar pattern of glycosylation between the Ch13-T14Y and CK2 peptides (Figs. 2 and 3).

Fig. 4 (*E* and *F*) shows the relative movement of individual backbone positions from 7 to 14 of the HCF-1_PRO_-repeat peptides (position 10 being the glycosylation or cleavage position) relative to the catalytic (*E*) and TPR (*F*) domains. The three HCF-1 threonine-rich region–containing peptides behaved nearly identically—relative to the catalytic domain, there was little change in motion across the eight residues (Fig. 4*E*). Relative to the TPR domain, residues 7–10 exhibited more motion than residues 11–14, which are closer to the TPR domain (Fig. 4*F*). In contrast, the CK2 peptide residues displayed a bipartite pattern relative to the catalytic domain, with the glycosylation region residues 7–11 moving considerably less than the C-terminal residues 12–14 (Fig. 4*E*), which are not anchored to OGT via the TPR domain by the threonine-rich region. The same decoupling of the CK2 peptide movements from the TPR movements can be observed in Fig. 4*F*. These results are consistent with the HCF-1_PRO_-repeat threonine-rich region serving to anchor peptides to OGT; such anchoring could explain the relaxed co-substrate requirements of the HCF-1_PRO_-repeat threonine-rich region–containing peptides.

### UDP-5S-GlcNAc is active for HCF-1_PRO_-repeat threonine-rich region containing peptide glycosylation

To probe further the effects of the threonine-rich region on UDP-GlcNAc co-substrate requirements, we tested the glycosylation substrate activities of the various peptides with the previously described 5S-GlcNAc thio-derivative of UDP-GlcNAc, a potent inhibitor of OGT glycosylation (6). For these experiments, we compared by LC-MS the levels of peptide glycosylation using the UDP-GlcNAc and UDP-5S-GlcNAc co-substrates. Owing to the molecular differences (hydrophobicity and molecular weight) of GlcNAc and 5S-GlcNAc, the glycosylated peptides could be distinguished from one another in both the HPLC and MS steps of the analysis, respectively.

**Figure 5.**
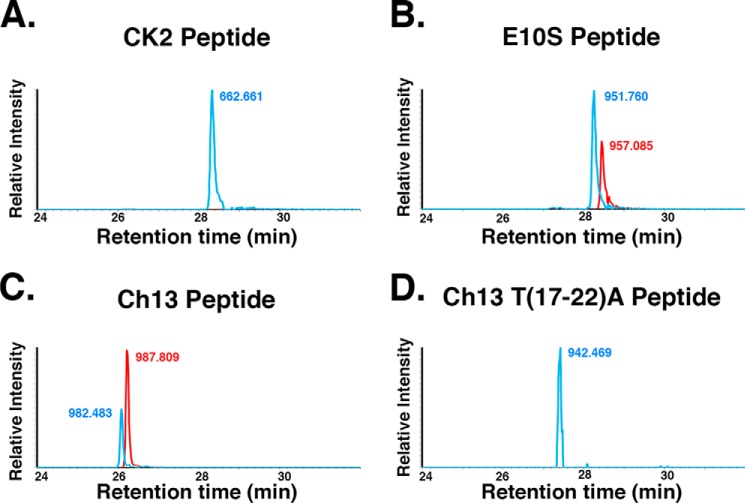
**UDP-5S-GlcNAc modification of HCF-1_PRO_-repeat threonine-rich region–containing peptides.** CK2 (*A*), HCF-1_PRO_-repeat 2 E10S (*B*), Ch13 (*C*), and Ch13 T(17–22)A peptides (*D*) were incubated with WT OGT and either UDP-GlcNAc (*blue*) or UDP-5S-GlcNAc (*red*). Peptide glycosylation was detected using LC-MS. The highest peak in each pair of samples was set at 100%. Single representative examples of three independent experiments are shown.

As expected, the CK2 peptide was only glycosylated by the UDP-GlcNAc co-substrate (Fig. 5*A*; *m*/*z* 662.661, *blue*). In contrast, the HCF-1_PRO_-repeat E10S peptide was effectively glycosylated with both the UDP-GlcNAc (*m*/*z* 951.760, *blue*) and UDP-5S-GlcNAc (*m*/*z* 957.085, *red*) co-substrates (Fig. 5*B*). The fusion of the HCF-1_PRO_-repeat threonine-rich region to the CK2 glycosylation region in a Ch13 peptide setting robustly activated UDP-5S-GlcNAc modification of the CK2 sequence (Fig. 5*C*, compare GlcNAc *m*/*z* 982.483 (*blue*) and 5S-GlcNAc *m*/*z* 987.809 (red)), which depended on the conserved threonine-rich region (Fig. 5*D*, *m*/*z* 942.469, *blue*). Thus, as for the *R*_p_-αS-UDP-GlcNAc diastereomer, the HCF-1_PRO_-repeat threonine-rich region relaxes the constraints on UDP-5S-GlcNAc co-substrate utilization.

### Activation of glutamate-directed cleavage in a heterologous context by the HCF-1_PRO_-repeat threonine-rich region

The interaction of OGT with the HCF-1_PRO_ repeat was first elucidated in the context of its unexpected role in cleavage of HCF-1 (1). Here, we have shown that the HCF-1_PRO_ repeat and in particular its threonine-rich region can activate unexpected enzymatic OGT glycosylation activities in CK2-HCF-1_PRO_-repeat chimeras. In so doing, we observed, as shown in Fig. 4*C*, that the structure of the CK2 glycosylation sequence in the Ch13 chimera can adopt a very similar backbone structure to the corresponding cleavage site and surrounding sequences of the OGT-bound HCF-1_PRO_ repeat. This led us to ask whether simple replacement of the glycosylated CK2 serine residue by a glutamate (*i.e.* S10E) might be sufficient to activate cleavage of this non-HCF-1 sequence.

**Figure 6.**
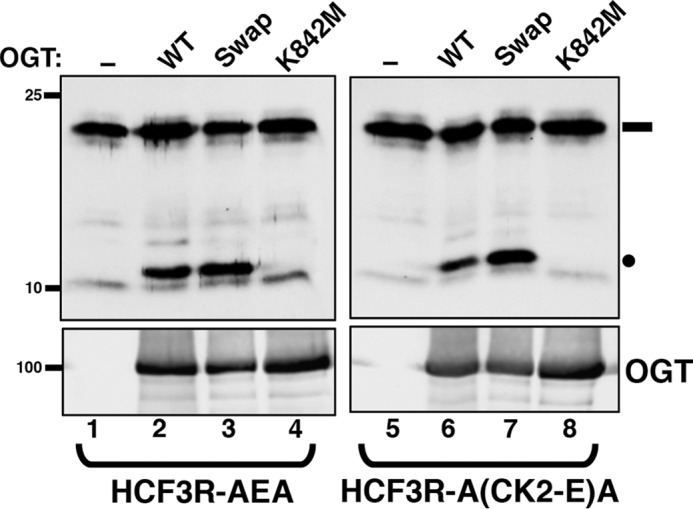
**Cleavage of chimeric glutamate modified CK2 sequence by OGT.** HCF3R-AEA (*lanes 1–4*) or chimeric HCF3R-A(CK2-E)A (*lanes 5–8*) protein were incubated without (*lanes 1* and *5*) or with either WT OGT (*lanes 2* and *6*) or OGT Swap (*lanes 3* and *7*) or K842M (*lanes 4* and *8*) mutant OGTs. HCF3R protein cleavage was assayed by SDS-PAGE and immunoblotting using an anti-His antibody. Anti-OGT was used to detect levels of OGT protein. *Black rectangle*, uncleaved HCF3R substrate; *black circle*, cleaved product.

To answer this question, we first mutated the three-HCF-1_PRO_ repeat–containing HCF3R cleavage substrate into a single-cleavage-site substrate by inactivating HCF-1_PRO_ repeats 1 and 3, creating HCF3R-AEA (see Table S2 for sequences). We then converted positions 3–13 of the central HCF-1_PRO_ repeat 2 into the corresponding CK2 glycosylation site sequence—creating a robust glycosylation substrate (called HCF3R-A(CK2-S)A), as shown in Fig. S2—followed by mutation of the single CK2 serine glycosylation acceptor into a glutamate residue, thus creating HCF3R-A(CK2-E)A. Fig. 6 shows the results of a cleavage assay with the HCF3R-AEA and HCF3R-A(CK2-E)A substrates. As expected, the WT HCF-1_PRO_ repeat in HCF3R-AEA was effectively cleaved by WT OGT (compare *lanes 1* and *2*) but not the inactive mutant K842M (*lane 4*) (2). A previously described glycosylation-defective but proteolysis-competent “Swap” mutant (D554H/H558D) (9) was, as expected, also active for cleavage (*lane 3*). The HCF3R-A(CK2-E)A substrate was also effectively cleaved (compare *lanes 5–8*), showing that OGT can cleave a substrate containing a glutamate residue at the right position (position 10) in a non-HCF-1 sequence context. The elevated cleavage activity observed with the OGT Swap mutant, in which residue Asp-554 is mutated, is consistent with the results of Janetzko *et al.* (3).

### HCF-1_PRO_-repeat threonine-rich region activates cleavage at a properly positioned glutamate residue

In the aforementioned experiments, only two prominent elements of the HCF-1_PRO_ repeat remained in the OGT proteolysis substrate: the glutamate cleavage-site residue at position 10 and the threonine-rich region. To address the importance of these two HCF-1_PRO_-repeat sequence elements for cleavage, we turned to the peptide and LC-MS–based cleavage assay recently described by Janetzko *et al.* (3), allowing us to simultaneously measure cleavage and glycosylation activities. An essential feature of the Janetzko *et al.* (3) peptide-based LC-MS cleavage assay was the identification of an extended HCF-1_PRO_-repeat peptide substrate (called HCF-short; see Table 1) that is efficiently cleaved by OGT into N- and C-terminal fragments of which the C-terminal fragment contains an N-terminal pyroglutamate cap (2, 3).

**Table 1:**
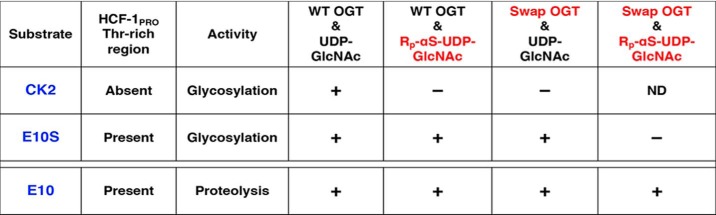
**Summary of glycosylation and proteolysis activities of HCF-1_PRO_ repeat–related and –unrelated substrates**

**Figure 7.**
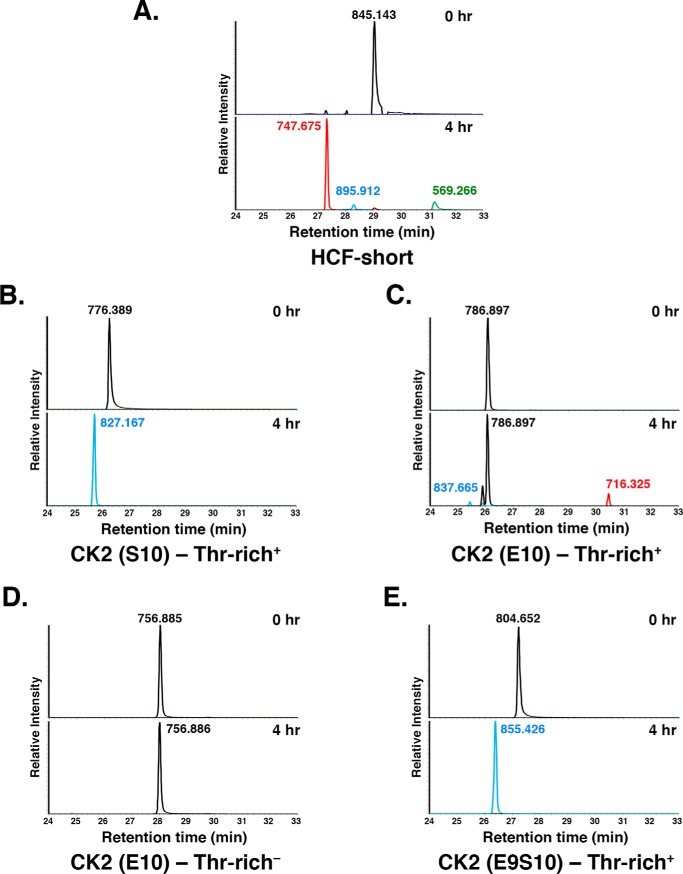
**Cleavage of chimeric glutamate–modified CK2 sequence by OGT requires the threonine-rich region and glutamate residue on position 10.** *A–E*, LC-MS analysis of WT OGT cleavage of the HCF-short (*A*), CK2 (Ser-10)–Thr-rich^+^ (*B*), CK2 (Glu-10)–Thr-rich^+^ (*C*), CK2 (Glu-10)–Thr-rich^−^ (*D*), and CK2 (E9S10)–Thr-rich^+^ (*E*) peptides at 0 and 4 h. Native (*black*) and glycosylated (*blue*) full-length peptides and cleaved N-terminal pyroglutamate-modified (*red*) and C-terminal (*green*) fragments are shown. The *y axis* in all plots is set at 100% for the tallest peak. In *C*, for the CK2 (Glu-10)–Thr-rich^+^ peptide, the tallest peak at the 4 h time point is ∼50% of the abundance level as compared with the 0 h time point. Single representative examples of three independent experiments are shown.

This HCF-short peptide was also effectively cleaved in our assay as shown in Fig. 7*A*. The starting peptide (*m*/*z* 845.143, *top*) essentially disappeared (*bottom*) after a 4-h incubation with OGT. In its place appeared N-terminal (*m*/*z* 569.266, *green*) and C-terminal pyroglutamate-capped (*m*/*z* 747.675, *red*) fragments. (The differing intensities of the cleaved peptides are probably owing to different detection efficiencies in MS.) We also observed a peptide (*m*/*z* 895.912, *blue*) corresponding to a monoglycosylated starting peptide; we did not map the site of this glycosylation, but its sensitivity to the *R*_p_-αS-UDP-GlcNAc diastereomer suggests that it is threonine, as opposed to glutamate, glycosylation (data not shown).

Using the HCF-short peptide as a template, we synthesized two CK2-HCF-1_PRO_ chimera peptides analogous to those used in Fig. 6 containing the CK2-Ser-10 glycosylation or CK2-Glu-10 cleavage sequence linked to the threonine-rich region of the HCF-1_PRO_ repeat (called CK2(Ser-10)–Thr-rich^+^ and CK2(Glu-10)–Thr-rich^+^, respectively; see Table S1). As shown in Fig. 7*B*, incubation of the CK2(Ser-10)–Thr-rich^+^ peptide with OGT resulted in the disappearance of the starting peptide (*m*/*z* 776.389) and conversion to a glycosylated peptide (*m*/*z* 827.167). Treatment of the CK2(Glu-10)–Thr-rich^+^ peptide (*m*/*z* 786.897) with OGT led to a 50% loss of CK2(Glu-10)–Thr-rich^+^ starting peptide (see the legend to Fig. 7). In its place, we observed a pyroglutamate-capped C-terminal fragment (*m*/*z* 716.325) and minor monoglycosylated starting product (*m*/*z* 837.665). The CK2 N-terminal cleavage fragment, which contains many hydrophilic residues, could not be detected. These results indicate that the CK2(Glu-10)–Thr-rich^+^ peptide is also a cleavage substrate of OGT but less effective than the parental HCF-short peptide.

Using this cleavable CK2(Glu-10)–Thr-rich^+^-peptide substrate, we first asked whether the threonine-rich region is important for cleavage activity by creating the CK2(Glu-10)–Thr-rich^−^ peptide substrate as shown in Fig. 7*D*. Indeed, this peptide (*m*/*z* 756.885/6) was not cleaved (nor glycosylated), showing the key importance of the TPR-bound threonine-rich region for activation of a glutamate-driven cleavage of a heterologous sequence.

The cleavable CK2(Glu-10)–Thr-rich^+^ substrate contains a C-terminal serine residue at position 11 (*i.e.* VE^10^S^11^), which is largely ignored (Fig. 7*D*). We asked here how the position of the glutamate (position 9 or 10) and serine (position 10 or 11) residues with respect to the threonine-rich region can influence the substrate cleavage and glycosylation activities of OGT. For this purpose, we inserted an additional alanine residue in the SAN linker sequence (changing the register with respect to the threonine-rich region of the VE^10^S^11^A^12^N sequence into an E^9^S^10^A^11^ A^12^N sequence; added alanine residue underlined), effectively “pushing” the glutamate and serine at positions 10 and 11 away from the C-terminal threonine-rich region to positions 9 and 10, respectively. This substrate, called CK2(E9S10)–Thr-rich^+^, was resistant to cleavage but was very effectively glycosylated (Fig. 7*E*), showing the importance of the three-amino acid linker region “register” between the TPR-bound threonine-rich region for defining either glutamate-driven cleavage or serine-driven glycosylation of an HCF-1_PRO_ repeat–related peptide substrate.

### E10S glycosylation, but not Glu-10 cleavage, is inhibited by combined disruption of the OGT enzyme and the UDP-GlcNAc co-substrate

To date, two mechanisms of OGT-induced glycosylation, which differ in how the serine/threonine acceptor site is deprotonated, have been described. (i) Schimpl *et al.* (5) propose acceptor deprotonation by the UDP-GlcNAc α-phosphate pro-R_P_ oxygen, an activity that would be inhibited by the *R*_p_-αS-UDP-GlcNAc diastereomer, and (ii) Lazarus *et al.* (12) propose a Grotthuss mechanism in which proton shuttling via water molecules occurs between the acceptor residue and OGT residue Asp-554. In support of UDP-GlcNAc α-phosphate pro-R_P_ oxygen deprotonation, the *R*_p_-αS-UDP-GlcNAc diastereomer is indeed inactive for normal substrate glycosylation (5, 9), and, consistent with a Grotthuss mechanism, mutation of the Asp-554 residue impairs normal substrate glycosylation (9).

**Figure 8.**
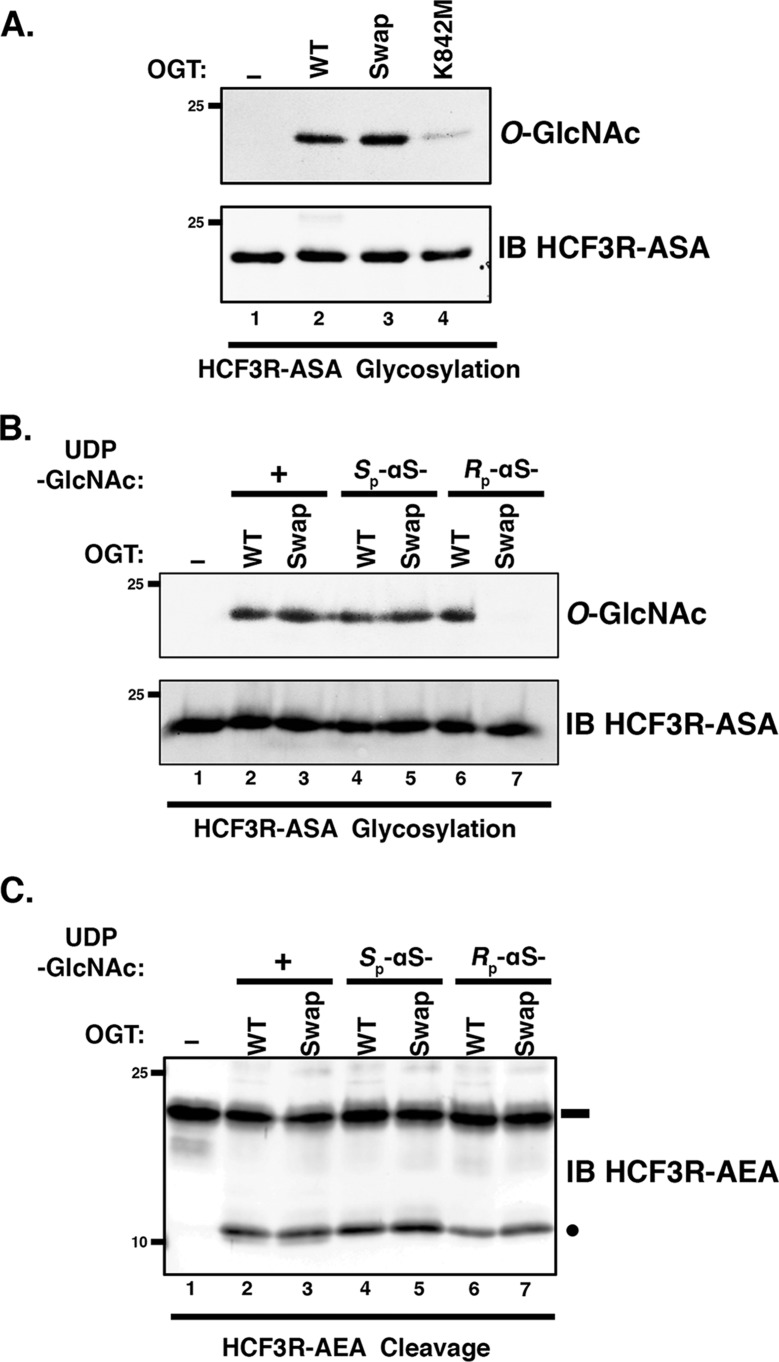
**OGT Swap mutant displays *R*_p_-αS-UDP-GlcNAc diastereomer co-substrate sensitivity for E10S glycosylation.** *A*, *in vitro* glycosylation of HCF3R-ASA substrate was assayed using either no OGT (*lane 1*) or WT (*lane 2*) or mutant Swap (*lane 3*) or K842M (*lane 4*) OGT. Substrate glycosylation was detected using RL2 anti-*O*-GlcNAc antibody. HCF3R-ASA protein (*IB*) was detected with anti-His antibody. *B*, *in vitro* glycosylation of HCF3R-ASA was assayed using either no OGT (*lane 1*) or WT or Swap OGT with 0.5 mm UDP-GlcNAc (*lanes 2* and *3*), *S*_p_-αS-UDP-GlcNAc (*lanes 4* and *5*), or *R*_p_-αS-UDP-GlcNAc (*lanes 6* and *7*). Substrate glycosylation was detected using RL2 anti-*O*-GlcNAc antibody. Anti-His antibody (*IB*) was used to detect the HCF3R-ASA protein. *C*, *in vitro* cleavage assay of HCF3R-AEA. The assay conditions are as in *B*. Anti-His antibody was used to detect uncleaved and cleaved HCF3R-ASA proteins. *Black rectangle*, uncleaved HCF3R substrate; *black circle*, cleaved product.

Given the unexpected activity of the *R*_p_-αS-UDP-GlcNAc diastereomer with the E10S HCF-1_PRO_ repeat, we decided to examine the activity of OGT mutants defective for normal substrate glycosylation, in particular the Swap mutant mutated at residue Asp-554 but active for proteolysis and the K842M mutant defective for both glycosylation and proteolysis (see Fig. 6), on an E10S HCF-1_PRO_-repeat substrate, as shown in Fig. 8*A*. Indeed, the otherwise glycosylation-defective OGT Swap mutant (9) was readily active on the E10S HCF-1_PRO_-repeat HCF3R-ASA substrate (compare *lanes 2* and *3*) and even displayed residual activity with the K842M mutant (*lane 4*). Thus, E10S glycosylation can occur with either the *R*_p_-αS-UDP-GlcNAc diastereomer, bypassing the α-phosphate pro-R_P_ oxygen deprotonation pathway, and with the Swap mutant bypassing a Asp-554–promoted Grotthuss mechanism.

To determine whether the remaining activities in these two different UDP-GlcNAc co-substrate and mutant OGT combinations are completely independent, we tested glycosylation activity in the presence of both the *R*_p_-αS-UDP-GlcNAc diastereomer and OGT Swap mutant as shown in Fig. 8*B*. Interestingly, whereas when tested separately (*lanes 2–6*), they were active on the E10S HCF3R-ASA substrate, when combined, the *R*_p_-αS-UDP-GlcNAc diastereomer and OGT Swap mutant were inactive on the E10S HCF3R-ASA substrate (*lane 7*). Thus, for glycosylation, the E10S HCF-1_PRO_-repeat substrate independently bypasses a requirement for the α-phosphate pro-R_P_ oxygen or for the Asp-554 residue implicated in the proposed Grotthuss mechanism but not both in combination.

Similar to these E10S glycosylation results, we have previously shown that the Glu-10 HCF-1_PRO_-repeat proteolysis substrate is active with either the *R*_p_-αS-UDP-GlcNAc diastereomer co-substrate or the OGT Swap mutant, but we had not tested them together for proteolysis (9). Fig. 8*C* shows such a test. Interestingly, in contrast to the E10S serine glycosylation substrate, the Glu-10 substrate is active for proteolysis in the presence of both the *R*_p_-αS-UDP-GlcNAc diastereomer co-substrate and the OGT Swap mutant (compare *lanes 2–6* and *7*). Such a result could be explained if proteolysis does not require assisted deprotonation of the target glutamate for its glycosylation and subsequent spontaneous backbone cleavage.

## Discussion

This study reveals two unusual OGT enzymatic activities in the context of the threonine-rich region of the HCF-1_PRO_ repeat: (i) enhanced serine glycosylation in the presence of either otherwise inefficiently used UDP-GlcNAc co-substrates or an OGT Swap mutant and (ii) glutamate-induced proteolysis of a novel sequence otherwise unrelated to HCF-1. In the first instance, high levels of serine glycosylation are achieved with the *R*_p_-αS-UDP-GlcNAc diastereomer and UDP-5S-GlcNAc–modified co-substrates or the otherwise inactive OGT Swap mutant, and in the second instance, a heterologous CK2 sequence possessing an appropriately positioned glutamate (Ser-to-Glu mutation) serves as a proteolytic substrate. Both unusual activities depend on the HCF-1_PRO_-repeat region—a region that anchors the protein substrate to the OGT TPR domain for “presentation” to the OGT catalytic domain. Thus, this study extends that of Kapuria *et al.* (9) in two important ways; it shows that the HCF-1_PRO_-repeat region can (i) activate unusual OGT serine glycosylation properties and not just proteolytic properties and (ii) promote proteolytic cleavage at a glutamate residue in a foreign cleavage-site context. Table 1 shows a summary of these unusual threonine-rich region–dependent activities. Glycosylation of a canonical, nonthreonine-rich region–containing substrate, represented here by CK2, was, as expected (5, 9), sensitive to both inactivation of a UDP-GlcNAc α-phosphate pathway with *R*_p_-αS-UDP-GlcNAc or an OGT-based mechanism with the OGT Swap mutant potentially inactivating a Grotthuss mechanism. In sharp contrast, however, an HCF-1_PRO_-repeat threonine-rich region–based substrate, here E10S, could circumvent inhibition of either pathway separately but, strikingly, not together. Thus, OGT and its UDP-GlcNAc co-substrate can apparently use two independent pathways for serine glycosylation, one enzyme-based (a Grotthuss pathway) and the other co-substrate–based (the α-phosphate pathway). Although an MD simulation study has suggested that an α-phosphate–based mechanism is more favorable than a Grotthuss-based mechanism (13), our study, although showing that each can occur independently, does not differentiate between the relative importance or favorability of the two pathways. Indeed, with the canonical CK2 substrate, where inhibition of either pathway inhibits glycosylation, the two likely cooperate for serine/threonine glycosylation. This multiplicity of OGT glycosylation strategies could represent targets of alternate regulatory strategies.

As mentioned previously, the unusual OGT glycosylation activities described here are dependent on the HCF-1_PRO_-repeat threonine-rich region. What is it about the threonine-rich region that activates such unusual activities? The threonine-rich region of the HCF-1_PRO_ repeat is unusual in how it forms an intimate association with the OGT TPR region (2) and promotes stable association with OGT (1, 11). In contrast, it does not seem to change how the site of glycosylation (or proteolysis) is presented to the OGT catalytic domain and its bound UDP-GlcNAc co-substrate. Thus, perhaps, it is the threonine-rich region–based stabilization of substrates on OGT that is providing sufficient conformational stability and/or time for independent utilization of either α-phosphate or Grotthuss pathways, which otherwise may be too inefficient for significant levels of glycosylation. Consistent with a stabilization hypothesis, the threonine-rich region also overcomes the low activity observed with the UDP-5S-GlcNAc co-substrate.

Interestingly, the intimate interaction of the HCF-1_PRO_-repeat threonine-rich region with the OGT TPR domain can be replaced by covalent fusion of an unrelated substrate to OGT at the TPR domain. The resulting fusion generated an efficient glycosylation substrate dependent on TPR domain interactions but not a threonine-rich region (14).

### Activation of proteolysis in a non-HCF-1_PRO_-repeat target sequence

HCF-1_PRO_-repeat proteolysis is exquisitely dependent on the HCF-1_PRO_-repeat threonine-rich region, yet it is remarkably resistant to alterations in OGT or the UDP-GlcNAc co-substrate that prevent both the proposed α-phosphate and Grotthuss serine glycosylation pathways (Table 1). Apparently, glutamate glycosylation, which is required for proteolysis (3), is not dependent on these two pathways. Perhaps, it is simply the stable association and proper positioning of the glutamate residue adjacent to the UDP-GlcNAc co-substrate that are prerequisites to HCF-1_PRO_-repeat glutamate glycosylation and subsequent cleavage. Consistent with this is the proteolytic activation shown here of a foreign sequence—the CK2 glycosylation sequence—for proteolysis.

Cleavage of the HCF-1_PRO_ repeat by OGT was a surprise (1). To date, it is the only known natural proteolytic target of OGT. Nevertheless, by making chimeric CK2/HCF-1_PRO_-repeat threonine-rich region peptides and simply converting the CK2 serine acceptor into a glutamate, we generated a sequence unrelated to the HCF-1_PRO_-repeat cleavage site that is cleaved by OGT. The position of the glutamate residue relative to the threonine-rich region was nevertheless critical. Thus, any other proteolytic targets of OGT would likely need to consist of a TPR domain–binding site with HCF-1_PRO_-repeat threonine-rich region properties followed by an appropriately positioned glutamate residue. Such other natural sites remain to be found.

## Experimental procedures

### Reagents and peptides

UDP-GlcNAc (U4375) was obtained from Sigma, whereas thio-analogues of UDP-GlcNAc (UDP-5S-GlcNAc, *S*_p_-αS-UDP-GlcNAc, and *R*_p_-αS-UDP-GlcNAc) were synthesized as described (5). Calf alkaline phosphatase was obtained from New England Biolabs. Peptides (Table S1) were obtained from Biomatik. Anti-OGT (sc-32921) and anti-CK2α (sc-12738) antibodies were purchased from Santa Cruz Biotechnology, Inc. (Dallas, TX), and anti*-O-*GlcNAc (RL2) antibody was purchased from Abcam (Cambridge, UK). Ni-NTA superflow and DTT were purchased from Sigma-Aldrich, and lysozyme was purchased from Roche Applied Science.

### HCF3R-vector construct preparation

cDNA sequences coding for HCF3R-ASA, -AEA, -A(CK2-E)A, and -A(CK2-S)A were synthesized by Eurofins MWG Operon (Ebersberg, Germany) and inserted into the pET47b vector digested with BamHI and NotI as described previously (9). The amino acid sequences are provided in Table S2. The fusion proteins contained a His_8_ tag at their N termini.

### Protein purification of OGT and HCF3R substrates

WT and mutant OGT and the AEA, ASA, A(CK2-E)A, and A(CK2-S)A HCF3R constructs were purified from *Escherichia coli* BL21(DE3) cells by Ni-NTA affinity as described previously (9). To concentrate and desalt the Ni-NTA eluate, Amicon concentration tubes (Millipore) were used (Ultra 3K for HCF3R, Ultra 10K for OGT) as per the manufacturer's instructions. The concentrated proteins were supplemented with 1 mm DTT and stored at −80 °C.

### Protein glycosylation assays

CK2α (2500 units; New England Biolabs) was incubated with OGT (WT; 500 nm) in 15 μl of TBS buffer supplemented with 0.1 mm UPD-GlcNAc, *S*_p_-αS-UDP-GlcNAc, or *R*_p_-αS-UDP-GlcNAc and 1 mm DTT at 37 °C for 60 min. The reaction was ended by the addition of 2× Laemmli buffer and boiling the sample. Anti*-O-*GlcNAc was used to assess the glycosylation of CK2, whereas anti-CK2 and anti-OGT were used to detect total CK2 and OGT protein levels, respectively. Similar assays was performed to probe HCF3R-ASA and HCF3R-A(CK2-S)A protein glycosylation using 25 μm substrate protein concentration and 0.5 μm OGT (WT, Swap, K842M). Anti-His tag antibody was used to detect total levels of HCF3R substrate proteins.

### Peptide glycosylation assays for LC-MS analyses

Peptide glycosylation assays were performed using 10 μm peptide, 25 μm UDP-GlcNAc, or thio-analogues of UDP-GlcNAc and 0.2 μm OGT in 20 μl of TBS buffer (50 mm Tris, pH 7.4, 150 mm NaCl) supplemented with 1 mm DTT and calf alkaline phosphatase (0.1 unit/μl). The reaction was terminated after 1 h by the addition of 20 μl of cold methanol.

### Protein cleavage assays

The HCF3R-AEA and - A(CK2-E)A cleavage assay was performed by mixing 25 μm HCF3R substrate with 1.0 μm OGT (WT, Swap, K842M) in 30 μl of TBS buffer supplemented with 0.1 unit/μl calf alkaline phosphatase, 1 mm UPD-GlcNAc (unless otherwise noted), and 1 mm DTT for 6 h at 37 °C. The reaction was ended by the addition of 2× Laemmli buffer and sample boiling. Anti-His antibody was used to detect cleavage of HCF3R proteins.

### Peptide cleavage assays for LC-MS analyses

Peptide cleavage assays were performed as described before (3) with slight modifications. Typical cleavage assay mixture contained 5 μm substrate peptide in 20 μl of TBS buffer supplemented with 0.1 units/μl calf alkaline phosphatase, 5 mm DTT, and 1 mm UDP-GlcNAc. Enzyme OGT (5 μm) was added last, and reactions were either terminated immediately (0 h time point) or after 4 h of incubation at 37 °C in a thermomixer, by the addition of 20 μl of cold methanol supplemented with 0.1% formic acid.

### LC-MS analyses

Peptide samples were diluted in a loading buffer (98:2 H_2_O/acetonitrile + 0.05% TFA) and injected on an Ultimate RSLC 3000 nanoHPLC system (Dionex, Sunnyvale, CA) interfaced to a high-resolution mass spectrometer, QExactive Plus (Thermo Fisher, Bremen, Germany). Peptides were loaded onto a trapping microcolumn, Acclaim PepMap100 C18 (20 mm × 100-μm inner diameter, 5 μm; Dionex) before elution on an Easy Spray C18 PepMap column (50 cm × 75-μm inner diameter, 2 μm, 100 Å; Dionex) at a flow rate of 0.25 μl/min. A gradient from 4 to 76% acetonitrile in 0.1% formic acid was used for peptide separation (total time, 65 min). Full MS survey scans were performed with resolution of 70,000. In data-dependent acquisition controlled by Xcalibur version 4.0 software, the 10 most intense multiple-charge precursor ions detected in the full MS survey scan were selected for higher-energy collision-induced dissociation (HCD, normalized collision energy = 27%) and analysis in the Orbitrap at 17,500 resolution. An isolation window of 1.5 *m*/*z* units around the precursor was used, and selected ions were then dynamically excluded from further analysis for 60 s.

For a more accurate localization of the *O*-GlcNAcylation on the E10S peptide, some samples were further analyzed with targeted electron transfer dissociation (with HCD supplemental activation) (EThcD) MS/MS fragmentation on an Orbitrap Fusion Tribrid mass spectrometer (Thermo Fisher Scientific, Bremen, Germany) interfaced via a nanospray source to a Dionex RSLC 3000 nanoHPLC system (Dionex, Sunnyvale, CA). After loading, peptides were separated on a custom-packed nanocolumn (75-μm inner diameter × 40 cm, 1.8-μm particles, Reprosil Pur, Dr. Maisch) with a gradient from 4 to 76% acetonitrile in 0.1% formic acid at a flow rate of 0.25 μl/min in 65 min. Targeted MS/MS of the 4^+^ charged state of the *O*-GlcNAcylated E10S peptide (*m/z* 714.072) was performed at 15,000 resolution, using EThcD fragmentation, with a 100-ms reaction time (electron transfer dissociation) and a supplemental activation energy at 25% (HCD).

### MS data processing

On the one hand, LC-MS/MS files were analyzed manually with Xcalibur software to extract *m*/*z* intensities of expected peptides, and on the other hand, MS/MS spectra were searched using Mascot version 2.6 software (Matrix Science, London, UK) to confirm identifications with a custom database containing the peptide sequences.

Mascot searches were carried out with a parent ion tolerance of 10 ppm and a fragment ion mass tolerance of 0.02 Da, without any enzyme cleavage. Oxidation of methionine and *N*-acetylhexosamine modification of serine and threonine were specified as variable modifications. Additional peptide C-terminal amidation and cysteine dehydro (half of a disulfide bridge) modification were also considered, depending on the sequence of the analyzed peptides. In cleavage assays, formation of pyroglutamic acid from N-terminal glutamic acid was also specified as a variable modification in Mascot searches.

### Immunoblotting

Proteins were resolved using SDS-polyacrylamide gels and blotted onto nitrocellulose membranes. The following primary antibodies were used at the following dilutions: anti*-O-*GlcNAc RL2 (1:1000), anti-OGT (1:5000), and anti-CK2α (1:1000). IRDye 680 donkey anti-rabbit and IRDye 800 donkey anti-mouse were used at dilutions of 1:10,000. Blots were imaged using the LI-COR Odyssey IR imaging system (LI-COR, Lincoln, NE).

### Molecular dynamics simulations

Periodic-boundary MD simulations were carried out with GROMACS version 2016.4 (15, 16), using the all-atom CHARMM27 force field (17, 18) and the TIP3P water model (19). Electrostatic interactions were calculated with the Ewald particle-mesh method (20) with a grid spacing of 1.2 Å and a spline interpolation of order 4. A cut-off of 10 Å was applied for the real-space direct sum part of the Ewald sum and also for the van der Waals interactions. Dispersion corrections were applied to the energy. Bonds involving hydrogen atoms were constrained using the P-LINCS algorithm (16). The system was coupled to a Berendsen barostat with a relaxation time of 1 ps. The solute and the solvent were separately coupled to two thermostats (21), each with a relaxation time of 0.2 ps. The time integration step was set to 2 fs, the temperature to 300 K, and the pressure to 1 bar. A cubic simulation cell with an edge length of 120 Å was used to prevent direct interactions between periodic images, resulting in about 170,000 atoms per system. Sodium atoms were added to neutralize each system. Initial structures were optimized, heated from 0 to 300 K over a period of 0.1 ns, equilibrated for a further 0.9 ns restraining each solute nonhydrogen atom to its original position, and finally equilibrated for 0.5 ns without restraints before data collection. For each system, eight simulations were carried out for a total of 200 ns of production time, saving coordinates every 0.05 ns. For analysis, structural snapshots were superimposed either based on the backbone of the OGT catalytic domain (residues 467–1028) or of the TPR domain (residues 312–466) before calculating backbone RMSDs from the respective X-ray structure.

Five different systems were simulated: OGT with HCF-1_PRO_-repeat Glu-10 and E10S, OGT with CK2 peptide, and OGT with chimeric peptides Ch13 and Ch13-T14Y. Simulations in complex with HCF-1–derived peptides were based on the X-ray structure 4N3B (2), replacing UDP-5S-GlcNAc with UDP-GlcNAc and Gln-10 with Glu or Ser. Simulations in complex with the CK2 peptide were based on the X-ray structure 4GYY (12), replacing UDP-5S-GlcNAc with UDP-GlcNAc. Simulations in complex with chimeric peptides were based on the homology model (see below).

### Homology model for chimeric Ch13 peptide

The model of the Ch13 peptide in complex with OGT was obtained using the Modeler program (22, 23), version 9. Template experimental structures (*i.e.* PDB entries 3PE4 (10), 4GYY (12), 4N39 (2), 4N3A (2), 4N3B (2), and 4N3C (2)) were taken from the Protein Data Bank (24). 500 models were produced and ranked according to the modeler objective function. The model with the best modeler objective function score was retained for analysis.

## Author contributions

V. K. performed all experiments except as otherwise indicated. U. F. R. and V. Z. designed and performed the computational modeling. P. W. performed HPLC for the peptide cleavage and glycosylation assays. F. L. prepared HCF3R vector constructs. V. S. B. and D. M. F. v. A. synthesized the UDP-5S-GlcNAc and αS-UDP-GlcNAc diastereomers. V. K. and W. H. designed the non-computational experiments and prepared the manuscript with input from all authors.

## Supplementary Material

Supporting Information

## Footnotes

4 The abbreviations used are:

OGT: *O*-linked *N*-acetylglucosamine transferase
MD: molecular dynamics
TPR: tetratricopeptide repeat
CK2: casein kinase 2
RMSD: root mean square deviation
Ni-NTA: nickel-nitrilotriacetic acid
HCD: higher-energy collision-induced dissociation
PDB: Protein Data Bank
EThcD: electron transfer dissociation (with HCD supplemental activation).
