# Supplementary material for: The conserved threonine-rich region of the HCF-1PRO repeat activates promiscuous OGT:UDP-GlcNAc glycosylation and proteolysis activities
Source: J Biol Chem. 2018 Sep 17;293(46):17754–68. doi: 10.1074/jbc.RA118.004185 (PMC6240873; doi:10.1074/jbc.RA118.004185)
Supplement: Supporting Information [file supp_293_46_17754__index.html]

The conserved threonine-rich region of the HCF-1PRO repeat activates promiscuous OGT:UDP-GlcNAc glycosylation and proteolysis activities — HCF-1 induced OGT glycosylation and cleavage activities — The conserved threonine-rich region of the HCF-1PRO repeat activates promiscuous OGT:UDP-GlcNAc glycosylation and proteolysis activities — HCF-1–induced OGT glycosylation and cleavage activities — Supporting Information 

# The conserved threonine-rich region of the HCF-1PRO repeat activates promiscuous OGT:UDP-GlcNAc glycosylation and proteolysis activities

## Supporting Information

- Supplementary Figure 1 - Kinetics of E10S peptide glycosylation by Sp- and Rp-&#x03B1;S-UDP-GlcNAc.
- Supplementary Figure 2 - OGT mediated cleavage and glycosylation of HCF3R substrates.
- Supplementary Table 1 - Sequences of peptides used for glycosylation and cleavage assays
- Supplementary Table 2 - HCF3R protein sequences
- Supporting Information - Supporting Information
