# Supplementary figures and images for: The conserved threonine-rich region of the HCF-1PRO repeat activates promiscuous OGT:UDP-GlcNAc glycosylation and proteolysis activities
Source: J Biol Chem. 2018 Sep 17;293(46):17754–68. doi: 10.1074/jbc.RA118.004185 (PMC6240873; doi:10.1074/jbc.RA118.004185)

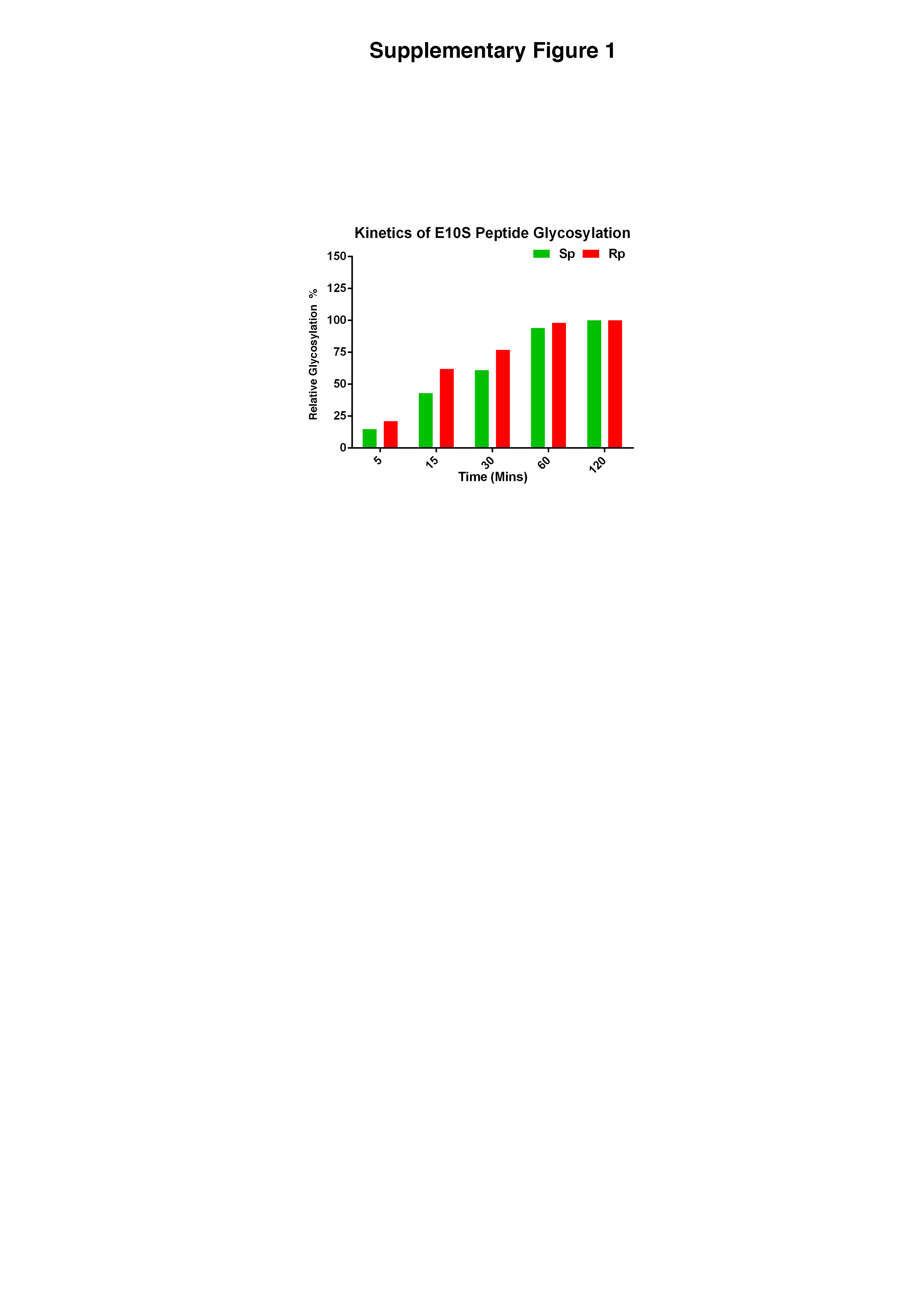

Supplement: Supporting Information [file supp_RA118.004185_138428_1_supp_193793_pp8lpk.tif]

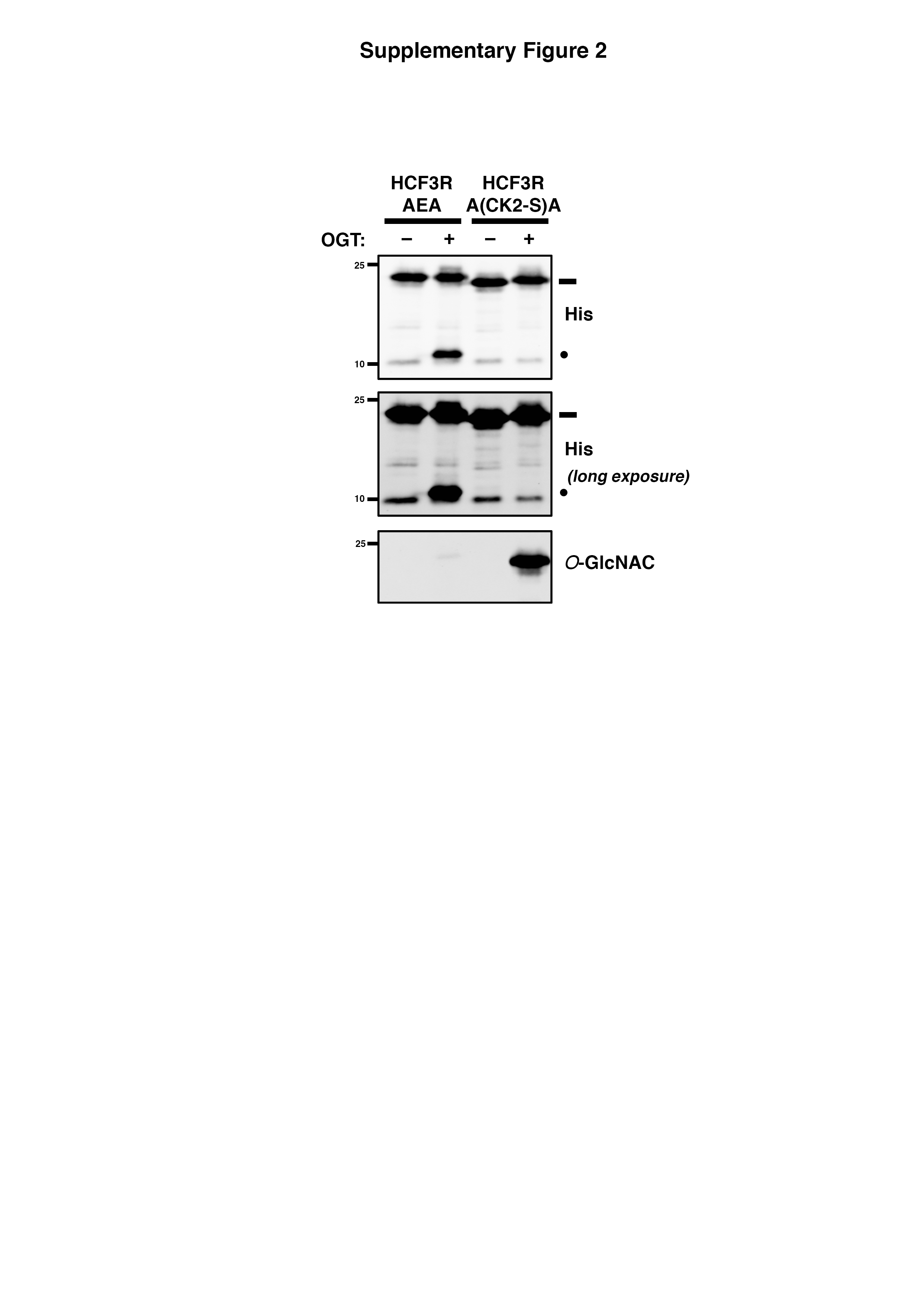

Supplement: Supporting Information [file supp_RA118.004185_138428_1_supp_193794_p78l7k.tif]

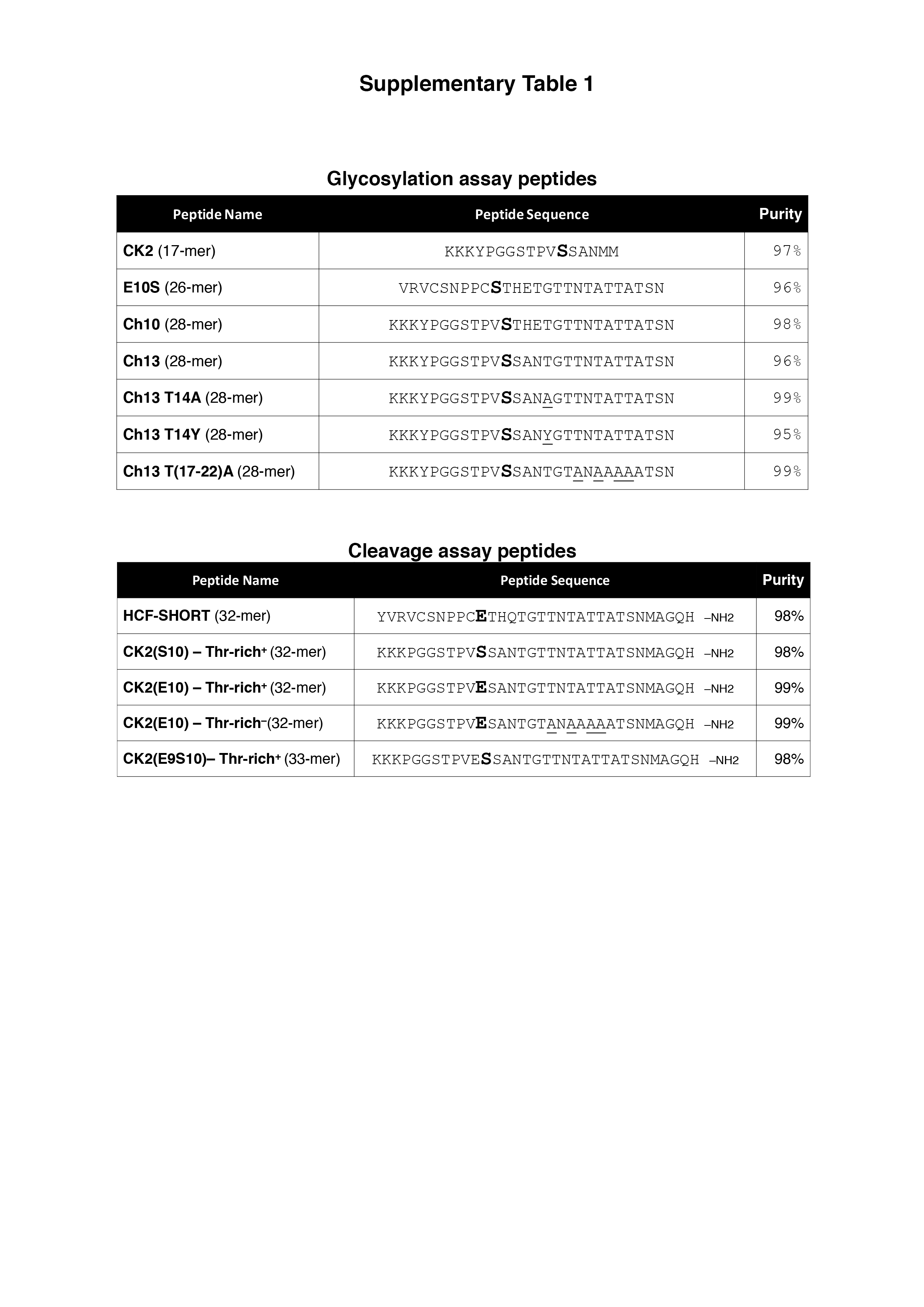

Supplement: Supporting Information [file supp_RA118.004185_138428_1_supp_193791_pv8lvk.tif]

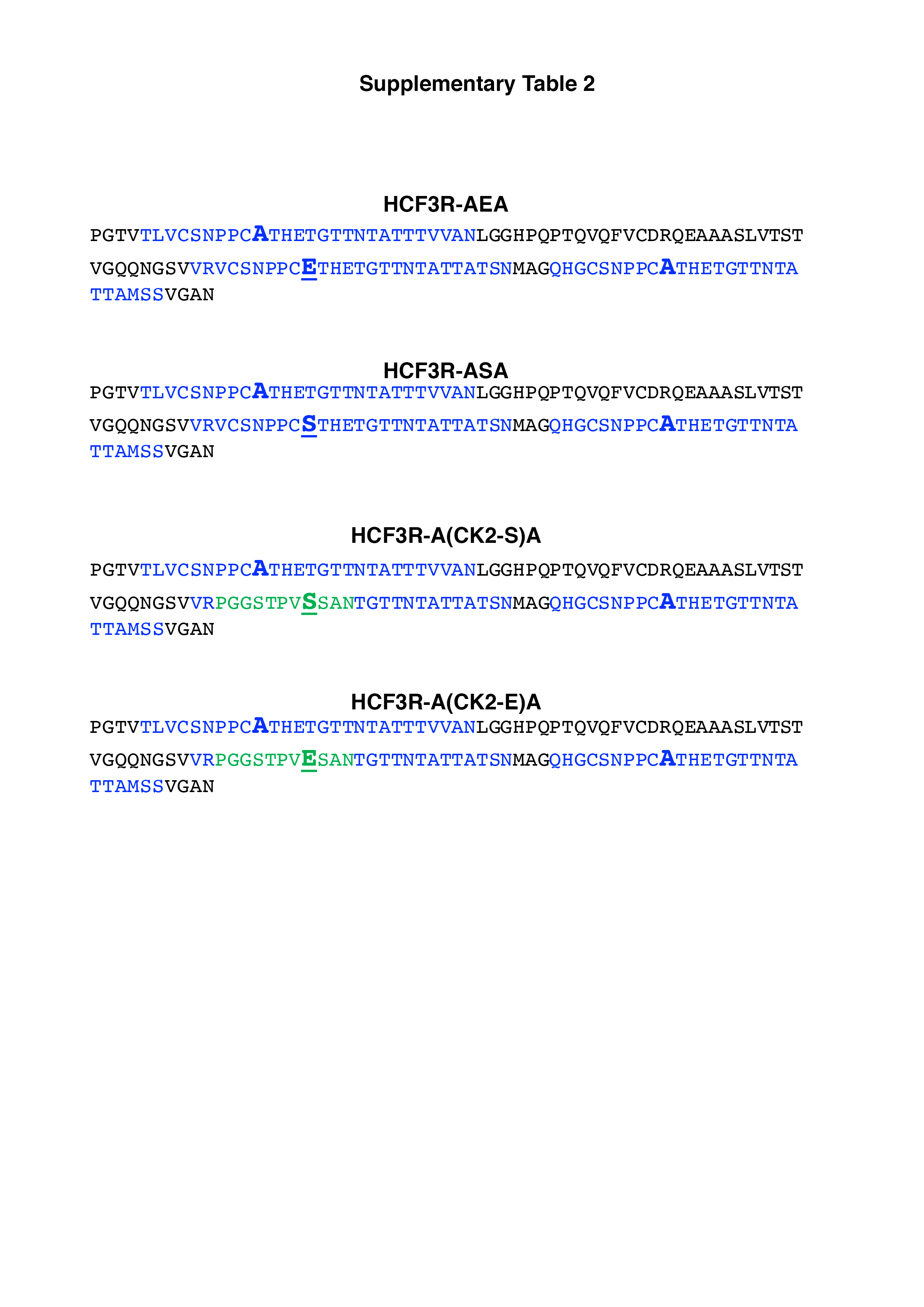

Supplement: Supporting Information [file supp_RA118.004185_138428_1_supp_193792_p98l9k.tif]
