# Supplementary material for: The conserved threonine-rich region of the HCF-1PRO repeat activates promiscuous OGT:UDP-GlcNAc glycosylation and proteolysis activities
Source: J Biol Chem. 2018 Sep 17;293(46):17754–68. doi: 10.1074/jbc.RA118.004185 (PMC6240873; doi:10.1074/jbc.RA118.004185)
Supplement: Supporting Information [file supp_RA118.004185_138428_1_supp_197527_pklk7y.pdf]

SUPPORTING INFORMATION

The conserved threonine-rich region of the HCF-1$_{PRO}$ repeat activates promiscuous OGT:UDP-GlcNAc glycosylation and proteolysis activities

Vaibhav Kapuria[1], Ute F. Röhrig[2], Patrice Waridel[3], Fabienne Lammers[1], Vladimir S. Borodkin[4], Daan M. F. van Aalten[4], Vincent Zoete[2,5], and Winship Herr[1]

From the [1] Center for Integrative Genomics, University of Lausanne, 1015 Lausanne, Switzerland; [2] Molecular Modelling Group, SIB Swiss Institute of Bioinformatics, Lausanne 1015, Switzerland.; [3] Protein Analysis Facility, Center for Integrative Genomics, Faculty of Biology and Medicine, University of Lausanne, 1015 Lausanne, Switzerland; [4] Centre for Gene Regulation and Expression, School of Life Sciences, University of Dundee, Dundee DD1 5EH, United Kingdom.; [5] Department of Fundamental Oncology, University of Lausanne, Ludwig Institute for Cancer Research, 1066 Epalinges, Switzerland.

Supplementary Figure 1

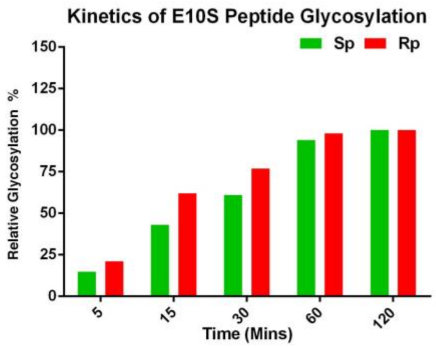

Supplementary Figure 1. Kinetics of E10S peptide glycosylation by S_p- and R_p-αS-UDP-GlcNAc.

In vitro peptide glycosylation assay was performed using HCF-1_{PRO}-repeat 2 E10S peptide. by incubating with WT OGT and either S_p-αS-UDP-GlcNAc (green bar) or R_p-αS-UDP-GlcNAc (red bar) as indicated. Samples were withdrawn at indicated time intervals and peptide glycosylation was detected using LC-MS as described in materials and methods. *O*-GlcNAcylation of the E10S peptide at different time points was normalized to the *O*-GlcNAcylation levels at 120 minute time point.

Supplementary Figure 2

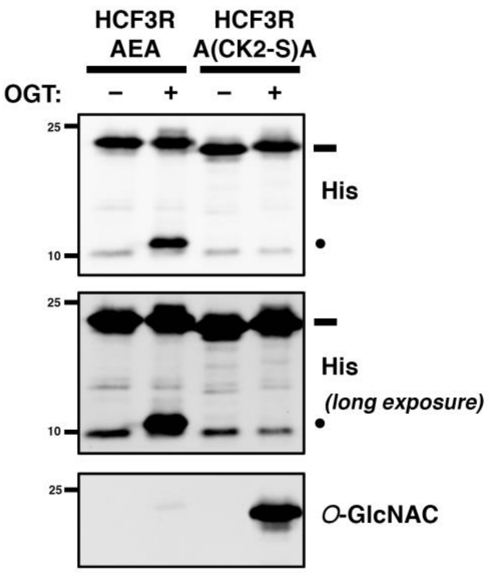

<u>Supplementary Figure 2.</u> OGT mediated cleavage and glycosylation of HCF3R substrates.

HCF3R-AEA or chimeric HCF3R-A(CK2-S)A protein were incubated with or without OGT for 6 hr. HCF3R protein cleavage and glycosylation was assayed by SDS-PAGE and immunoblotting using an anti-His antibody (for cleavage) and RL2 anti-*O*-GlcNAc antibody (glycosylation). Anti-OGT was used to detect levels of OGT protein. Chimeric HCF3R-A(CK2-S)A protein did not undergo any cleavage (lack of cleavage band in long exposure) but was heavily glycosylated as compared to native HCF3R-AEA substrate.
(▬, un-cleaved HCF3R substrate; ●, cleaved product.)

Supplementary Table 1

Glycosylation assay peptides

Peptide Name	Peptide Sequence	Purity
**CK2** (17-mer)	KKKYPGGSTPV**S**SANMM	97%
**E10S** (26-mer)	VRVCSNPPC**S**THETGTTNTATTATSN	96%
**Ch10** (28-mer)	KKKYPGGSTPV**S**THETGTTNTATTATSN	98%
**Ch13** (28-mer)	KKKYPGGSTPV**S**SANTGTTNTATTATSN	96%
**Ch13 T14A** (28-mer)	KKKYPGGSTPV**S**SANAGTTNTATTATSN	99%
**Ch13 T14Y** (28-mer)	KKKYPGGSTPV**S**SANYGTTNTATTATSN	95%
**Ch13 T(17-22)A** (28-mer)	KKKYPGGSTPV**S**SANTGTANAAAAATSN	99%

Cleavage assay peptides

Peptide Name	Peptide Sequence	Purity
**HCF-SHORT** (32-mer)	YVRVCSNPPC**E**THQTGTTNTATTATSNMAGQH –NH2	98%
**CK2(S10) – Thr-rich+** (32-mer)	KKKPGGSTPV**S**SANTGTTNTATTATSNMAGQH –NH2	98%
**CK2(E10) – Thr-rich+** (32-mer)	KKKPGGSTPV**E**SANTGTTNTATTATSNMAGQH –NH2	99%
**CK2(E10) – Thr-rich−**(32-mer)	KKKPGGSTPV**E**SANTGTANAAAAATSNMAGQH –NH2	99%
**CK2(E9S10)– Thr-rich+** (33-mer)	KKKPGGSTPVE**S**SANTGTTNTATTATSNMAGQH –NH2	98%

Supplementary Table 2

HCF3R-AEA

PGTVTLVCSNPPC**A**THETGTTNTATTTVVANLGGHPQPTQVQFVCDRQEAAASLVTST
VGQQNGSVVRVCSNPPC**E**THETGTTNTATTATSNMAGQHGCSNPPC**A**THETGTTNTA
TTAMSSVGAN

HCF3R-ASA

PGTVTLVCSNPPC**A**THETGTTNTATTTVVANLGGHPQPTQVQFVCDRQEAAASLVTST
VGQQNGSVVRVCSNPPC**S**THETGTTNTATTATSNMAGQHGCSNPPC**A**THETGTTNTA
TTAMSSVGAN

HCF3R-A(CK2-S)A

PGTVTLVCSNPPC**A**THETGTTNTATTTVVANLGGHPQPTQVQFVCDRQEAAASLVTST
VGQQNGSVVRPGGSTPV**S**SANTGTTNTATTATSNMAGQHGCSNPPC**A**THETGTTNTA
TTAMSSVGAN

HCF3R-A(CK2-E)A

PGTVTLVCSNPPC**A**THETGTTNTATTTVVANLGGHPQPTQVQFVCDRQEAAASLVTST
VGQQNGSVVRPGGSTPV**E**SANTGTTNTATTATSNMAGQHGCSNPPC**A**THETGTTNTA
TTAMSSVGAN
